# Differences in Central Corneal Thickness between Spectral Domain-Optical Coherence Tomography and Ultrasound Pachymetry in Patients with Dry Eye Disease

**DOI:** 10.1155/2016/2623719

**Published:** 2016-06-08

**Authors:** Ali Riza Cenk Celebi, G. Ertugrul Mirza

**Affiliations:** ^1^Department of Ophthalmology, Acibadem University School of Medicine, 34303 Istanbul, Turkey; ^2^Department of Ophthalmology, Erciyes University School of Medicine, 38030 Kayseri, Turkey

## Abstract

*Purpose*. To compare central corneal thickness (CCT) values via Spectral Domain-Optical Coherence Tomography (SD-OCT) and ultrasonic pachymetry in patients with severe dry eye disease (DED) to determine the level of agreement between these 2 methods.* Methods*. The paired samples *t*-test was used to compare CCT values in severe DED patients. Matching analysis between methods was performed using intraclass correlation coefficient (ICC). Intrasession reliability of the measurement methods was calculated via the concordance correlation coefficient (CCC), variation equivalent, and Pearson's correlation coefficient. The Bland-Altman procedure was used to graphically represent the differences between CCT values.* Results*. The study included 56 eyes of 24 female and 4 male patients. Mean age of the patients was 50.9 ± 11.3 years. Mean CCT via Cirrus SD-OCT was 523.82 ± 30.98 *μ*m versus 530.050 ± 31.85 *μ*m via ultrasonic pachymetry (paired samples *t*-test, *P* < 0.001). The Bland-Altman plot showed good agreement between the examiners. The ICC for repeatability was 0.974. The CCC between the 2 methods' CCT values was 0.973. The variation equivalent was 0.976 and Pearson's correlation coefficient was 99.3%, which also indicated high correlation between the 2 methods' measurements.* Conclusions*. The present findings show that in patients with severe DED Cirrus SD-OCT provides reliable intraobserver CCT values.

## 1. Introduction

Dry eye disease (DED) is a multifactorial disease that negatively affects tears and the ocular surface, resulting in potential corneal injury [1]. Epidemiological data have shown that dry eye becomes more frequent with age in both sexes. Women are at a higher risk of dry eye than men [2]. The mechanisms of DED include tear film instability, increased tear osmolarity, and a cascade of inflammatory events in the corneal epithelial surface [1]. In order to plan refractive surgery, detect corneal changes due to corneal disease, or measure intraocular pressure (IOP), it is important to measure corneal thickness precisely [3]. Evaluation of corneal thickness provides clinically useful information related to the physiological status of the cornea [4]. Significant alterations in central corneal thickness (CCT) have the potential to alter IOP measurement. Underestimation of IOP because of a thin cornea can potentially delay diagnosis and treatment of glaucoma [5]; therefore, evaluation of CCT is essential in cases of glaucoma, contact lens wear, corneal refractive surgery, and dry eye disease [4].

As CCT is an important indicator of corneal health, various studies have investigated CCT and dry eye disease—some reporting thin CT in patients with DED [6–9]. Furthermore, Karadayi et al. [9] suggested that CCT might be used for the diagnosis and follow-up of patients with DED. Reliable CCT measurement can be obtained using a variety of methods, including ultrasonic pachymetry, scanner slit technology, rotating Scheimpflug imaging, interferometry, corneal confocal microscopy, and optical coherence tomography (OCT) [10]. Ultrasonic pachymetry is currently the gold standard for measuring CCT; however, use of its contact probe is associated with patient discomfort and microbial contamination [10]. It was reported that patients with DED are more susceptible to corneal infections than healthy individuals [11]. Furthermore, ultrasonic pachymetry can easily produce corneal epithelial damage over the ocular surface which is much more severe in patients with DED and it can produce measurement error due to the pressure exerted over the cornea and due to inadequate alignment of the terminal, which must be positioned absolutely perpendicular to the corneal surface [12]. Because of these drawbacks associated with ultrasonic pachymetry, OCT has been recently applied to the measurement of CCT.

OCT was originally used to diagnose retinal pathologies. Technological advancements in OCT have made it possible to use Spectral Domain-Optical Coherence Tomography (SD-OCT) to image corneal tissue. The Cirrus SD-OCT device is among the latest generation of Fourier domain OCT devices and can imagine anterior segment structures by changing the focus of the OCT beam. Previous studies have evaluated the accuracy and reliability of SD-OCT measurement of CCT [10, 13]. In general, mean CCT values obtained via SD-OCT were lower than those obtained via ultrasonic pachymetry. In an earlier study of ours [13] mean CCT measured via SD-OCT was 3.37 *μ*m less than that via ultrasonic pachymetry. The reliability of measurements obtained using any ophthalmic instrument should be determined, so that misdiagnosis based on the readings can be avoided. To the best of our knowledge, the reliability of Cirrus SD-OCT measurement of CCT in patients with DED has not been studied; as such, the present study aimed to compare CCT measurement via SD-OCT and ultrasonic pachymetry in patients with DED to determine the degree of systematic difference and the level of agreement between the 2 methods. The possible mechanisms and theoretical explanations regarding the difference of CCT measurements obtained by SD-OCT and ultrasonic pachymetry in dry eye patients were also discussed. Therefore, it was also discussed that, in clinics using Cirrus SD-OCT as a diagnostic imaging method, this device could be used as a reliable noncontact pachymeter when assessing dry eye patients. This study also aimed to determine the intraexaminer reproducibility of CCT values via the two methods in patients with DED.

## 2. Materials and Methods

This prospective observational study included 28 patients diagnosed as severe DED. The study protocol was approved by the Local Ethical Committee and was performed in accordance with the Declaration of Helsinki, and each patient provided written informed consent for imaging and DED assessment at the time they visited our clinic. DED was diagnosed based on tear film breakup time with ocular surface staining <5 s, Schirmer's test result (with topical proparacaine anesthesia) <5 mm, and significant symptoms of dryness at presentation as confirmed with OSDI score which is over 40 [1]. Patients with a history of corneal surgery and those with evidence of active infection in the cornea and/or conjunctiva, localized corneal scar, and reported use of contact lenses were excluded from the study. Patients with any type of corneal dystrophy and/or rheumatic disease were also excluded as were those with a history of any ocular or systemic disease other than DED. All patients underwent comprehensive examination of the anterior and posterior segment structures using slit-lamp biomicroscopy, indirect fundoscopy, and applanation tonometry; patients with any type of posterior segment abnormality were also excluded from the study.

The central part of the cornea was found using calipers. The horizontal and the vertical diameter of the cornea were measured and the center of the distance was pointed. Both SD-OCT and ultrasonic measurements of the central corneal thicknesses were obtained from that central point.

CCT measurement via Cirrus SD-OCT was performed in each patient before CCT measurement via ultrasonic pachymetry, because ultrasonic pachymetry can cause corneal epithelial defects. Corneal images were acquired using the Cirrus SD-OCT device in anterior segment 5-line raster mode, which utilizes 5 horizontal scan lines—each 3 mm long—with a distance of 250 *μ*m between each two lines. Each scan line is composed of 4096 A scans s^−1^. This mode can easily image the upper (upper border of epithelia) and lower (inner border of endothelia) boundaries of the cornea with great clarity. In addition, the digital caliper can be placed very accurately between these boundaries.

After being seated and properly aligned in front of the device, each patient was instructed to focus on the device's internal fixation target during image acquisition. CCT anterior segment 5-line raster images were obtained for both eyes in each patient. Only images with signal strength ≥7 were evaluated. Examinations were performed between 12.00 and 13.00 to minimize the effect of diurnal variation in corneal thickness [9]. Among the CCT anterior segment 5-line raster images, the image at the center point of the cornea was enlarged. Then, CCT was measured via manual use of a digital caliper in the cross-line scan; the vertical distance between the inner border of endothelia and outer upper border of epithelia of the cornea was considered CCT. The measurements were carried out at the corneal center. CCT measurements were always manually performed at the corneal center point. Five consecutive measurements were obtained from each of the different Cirrus SD-OCT images and the mean CCT value was used for analysis. Immediately following CCT measurement via Cirrus SD-OCT, 1 drop of topical proparacaine 0.5% was placed in the same eye. Then, 5 measurements of the cornea were obtained using a PacScan 300P (Sonomed Escalon, Lake Success, NY, USA) ultrasonic pachymeter, with the ultrasonic probe at the center point of the cornea. Patients were instructed to fixate at a distant object, and then 5 consecutive measurements were obtained and averaged for comparison with Cirrus SD-OCT values.

Cirrus SD-OCT images were obtained and CCT values were calculated by the same physician who was blinded to the ultrasonic pachymetry CCT values to avoid bias. All ultrasonic pachymetry CCT measurements were made by the same ophthalmic technician to avoid interexaminer variability.

### 2.1. Statistical Analysis

Data were analyzed using SPSS v.16.0 for Windows (SPSS, Inc., Chicago, IL). Quantitative variables, such as CCT, were summarized using descriptive statistics (i.e., sample size frequency, percentage, mean, and standard deviation). Normality of data distribution was tested using the Shapiro-Wilk and Kolmogorov-Smirnov tests. The paired samples *t*-test was used to compare CCT values. The paired samples *t*-test was used to determine if there was a significant systematic bias between examiners. Matching analysis between both methods was performed using the intraclass correlation coefficient. Intrasession reliability of the measurement methods was calculated via the concordance correlation coefficient, intraclass correlation coefficient (ICC), variation equivalent, and Pearson's correlation coefficient, which was also used to investigate the correlation between the quantitative measurements of mean CCT.

The Bland-Altman procedure was used to graphically represent the differences between CCT values obtained via the 2 methods, as well as in the matching limits of the 95% limits of agreement (LoA). The 95% LoA was defined as the mean difference in measurements performed by the 2 examiners obtained by the 2 methods ±1.96 SD, with lower values indicating higher interobserver reproducibility. A Bland-Altman plot was generated to assess the difference in individual measurements as a function of the mean of 2 measurements and to evaluate the correlations between the 2 CCT measurement methods (MedCalc Software, Mariakerke, Belgium). In brief, agreement between the measurements obtained via the 2 methods was examined using a Bland-Altman plot and LoA were calculated. Pearson's correlation analysis was used to assess the strength of correlation between the measurements. Reproducibility was evaluated via the intraclass correlation coefficient (ICC); an ICC of 1.00 represents perfect agreement, whereas 0.81–0.99 represents almost perfect agreement. Results were evaluated at the 95% CI and the level of statistical significance was set at *P* < 0.05.

## 3. Results

The study included 56 eyes of 24 female and 4 male patients. Mean CCT value of the females via Cirrus SD-OCT was 520.67 ± 33.58 *μ*m versus 527.46 ± 35.07 *μ*m via ultrasonic pachymetry. Meanwhile the mean CCT value of the males via Cirrus SD-OCT was 533.00 ± 20.49 *μ*m versus 540.00 ± 20.94 *μ*m via ultrasonic pachymetry. These mean CCT value differences (6.79 *μ*m in females and 7 *μ*m in males) between Cirrus SD-OCT and ultrasonic pachymetry were significant in each sex (paired samples *t*-test, *P* < 0.001 for females and males). Indeed, the mean CCT value differences (12.33 *μ*m with Cirrus SD-OCT and 12.54 *μ*m with ultrasonic pachymetry) between sexes were substantially different (Mann-Whitney *U* test, *P* < 0.001).

Mean age of the patients was 50.9 ± 11.3 years. The mean age of female patients was 51.7 ± 11.4 years; however, the mean age for the males was 45.7 ± 10.1 years. All 28 patients had the same Schirmer values in both of their eyes individually.

Mean CCT value of the right eyes via Cirrus SD-OCT was 522.43 ± 32.04 *μ*m versus 529.25 ± 33.41 *μ*m via ultrasonic pachymetry. Meanwhile the mean CCT value of the left eyes via Cirrus SD-OCT was 525.21 ± 30.39 *μ*m versus 530.86 ± 30.79 *μ*m via ultrasonic pachymetry. The mean difference between the 2 methods in left and right eyes separately was statistically significant (paired samples *t*-test, *P* < 0.001 for left eyes; paired samples *t*-test, *P* < 0.001 for right eyes). However, the CCT values between eyes were not statistically significant (paired samples *t*-test, *P* > 0.05). Both eyes were used in analysis because the mean CCT values in right eyes and left eyes for each of the measurement devices showed high correlation [14]. The concordance correlation coefficients of the right eyes and left eyes were 0.97 and 0.98, respectively.

**Figure 1:**
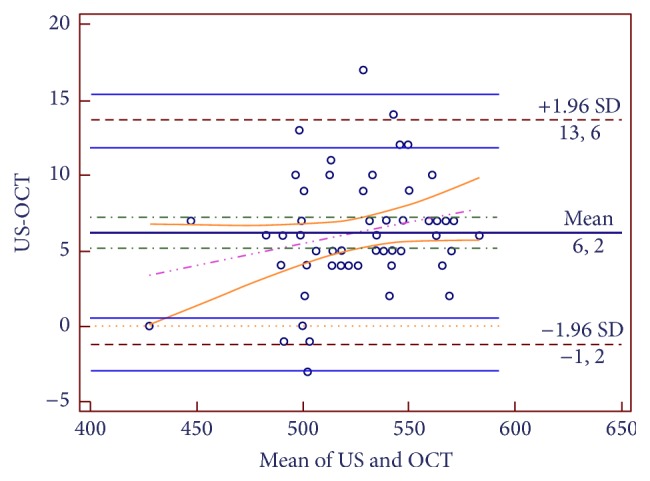
Bland-Altman plot of the difference in mean CCT values based on Cirrus SD-OCT and ultrasonic pachymetry in patients with DED.

Overall mean CCT via Cirrus SD-OCT was 523.82 ± 30.98 *μ*m versus 530.050 ± 31.85 *μ*m via ultrasonic pachymetry; the mean difference between the 2 methods' measurements was significant (6.23 *μ*m) (paired samples *t*-test, *P* < 0.001). The distribution of the differences between both methods' measurements was normal (Shapiro-Wilk test, *P* > 0.05). The Bland-Altman plot generated to assess the difference in individual measurement as a function of the mean of 2 measurements showed good agreement between the examiners; LoA width was 14.8 *μ*m. The upper limit of LoA was 13.64 (95% CI: 11.90–15.38) and the lower limit was −1.18 (−2.92–0.56) (Figure 1). The ICC for repeatability was 0.974 (95% CI: 0.955–0.984). The correlation coefficient (CCC) between the 2 methods' CCT measurements was 0.973 (95% CI: 0.958–0.983). In addition, the variation equivalent was 0.976 and Pearson's correlation coefficient was 99.3%, which also indicated high correlation between the 2 methods' measurements.

## 4. Discussion

Evaluation of corneal thickness provides clinically useful information concerning the physiological status of the cornea. Ultrasonic pachymetry has been the gold standard for measuring corneal thickness since 1967 based on a Pubmed search. The primary drawback of this technique is that it is invasive and requires instillation of topical anesthesia [15]. In addition, ultrasonic pachymetry is associated with several potential sources of error in terms of CCT measurement. Its accuracy depends on the cornea, and the perpendicularity of the probe with respect to cornea is often difficult to ascertain. If the probe is placed slightly off center at an oblique angle, corneal thickness can be overestimated. Due to these drawbacks associated with ultrasonic pachymetry, various noncontact methods of CCT measurement have recently come into use [16]. OCT is an in vivo, noncontact technique for obtaining high-resolution, cross-sectional images of biological tissues based on measurement of optical reflections. Recently, the utility of OCT in clinical practice has been extended to the anterior segment of the eye [16]. Moreover, dedicated noncontact SD-OCT devices have become available, offering rapid acquisition of high-resolution, cross-sectional images of the cornea and CCT measurement. Anterior segment optical coherence tomography (AS-OCT) is not associated with the same disadvantages as ultrasonic pachymetry, because it is a noninvasive, noncontact method. Earlier studies evaluated corneal thickness using the OCT intensity profile, in which computer software-controlled cursors are manually placed at the peak of reflectivity corresponding to the tissue interfaces [17]. In the present study, in consideration of the resolution of the OCT images obtained, we directly and manually placed the cursors provided by the SD-OCT software to measure CCT.

DED is a disease of the ocular surface that causes discomfort, visual disturbance, and tear film instability, with the possibility of damage to the ocular surface. DED is accompanied by an increase in osmolarity of the tear film and inflammation of the ocular surface [1]. As the assessment of CCT is an important indicator of corneal health, various studies have investigated CCT in patients with DED [9, 18–20]. Pole and Batzer [18] studied 16 patients with DED and observed a minimal, nonsignificant reduction in corneal thickness in the patients with DED, as compared to healthy controls, whereas Høvding [19] reported a significant reduction in CCT in the absence of marked inflammation in 17 patients with DED.

At the time those earlier studies were performed, ultrasonic pachymetry was the only available method for measuring CCT in patients with DED. A groundbreaking study reported a significant decrease in CCT measured via Orbscan pachymetry in patients with aqueous tear-deficient DED. The researchers measured CCT in 38 eyes of 21 patients with DED and in 34 eyes of 21 healthy controls, and the mean difference between the 2 groups was 35 *μ*m [6]. A more recent study that also used Orbscan pachymetry reported a reduction in CCT in postmenopausal women with DED [8].

The International Dry Eye Workshop [1] modified the definition of DED to include the role of tear film hyperosmolarity and ocular surface inflammation. Regardless of initiating etiological factors, once DED develops inflammation becomes the key mechanism of injury. Excessive inflammation can lead to a vicious cycle, resulting in an increase in tear film osmolarity. In all forms of DED the primary mechanism of ocular surface damage is apparently hyperosmolarity of the tear film, which affects corneal hydration resulting in dehydration of the cornea [20]. In healthy individuals, the aqueous layer of the tear film is isotonic or slightly hypertonic. Hypertonic solutions decrease corneal thickness and it was reported that when tear production decreases tear film osmolarity increases and the cornea becomes thinner [6].

The etiology of decreased corneal thickness in patients with DED is not clearly understood. Among the proposed etiological factors are an increase in tear film evaporation which results in increased osmolarity of the tear fluid, as mentioned above, and a chronic state of desiccation and immune activation that can cause a decrease in tear film thickness, which is normally 1–45 *μ*m [6–8]. It was suggested that drying conditions stimulate cells to cycle and proliferate throughout the entire epithelium as a consequence of apical cell surface damage [19]. In an experimentally induced DED model, Yeh et al. [21] observed apoptosis of keratocytes and suggested that apoptosis might play an important role in the pathogenesis of DED-related epitheliopathy. Hence, it was suggested that excessive apoptosis or shedding of the surface epithelium—if sustained and if there is no compensation for epithelial cycling—might lead to epithelial thinning in patients with DED [22]. Furthermore, a significant reduction in corneal stromal thickness in DED patients was observed via in vivo confocal microscopy. It was hypothesized that apoptosis, as well as an increase in proteolytic activity at the stromal level, might be the cause of this reduction in corneal stromal thickness [23]. An in vivo confocal study reported that the density of superficial corneal epithelial cells and subbasal nerves was significantly lower in patients with DED than in healthy individuals [23]. In addition, the density of superficial and intermediate epithelial cells at the center of the cornea in dry eye patients was less than that in healthy controls, which might have been due to enlargement of the cells as a result of metabolic dysfunction [23].

Another theoretical explanation for the difference in CCT values between ultrasonic pachymetry and SD-OCT methods is corneal edema due to topical anesthetic eye drops [24]. Topical ophthalmic anesthetics are toxic to the cornea; however, they are needed for a number of clinical diagnostic procedures, including ultrasonic pachymetry. The potential adverse effects of topical anesthetics include tear film alteration, epithelial toxicity, microbial contamination, and allergic reactions. Anesthetic eye drops can induce variations in corneal thickness >10 *μ*m, masking the possible effect of dry eye on CCT values in patients with DED. Herse and Siu [25] observed that instillation of a single drop of proparacaine 0.5% induced an increase in CCT which was caused by corneal stromal edema. Mukhopadhyay et al. [26] reported overall corneal swelling following administration of topical proparacaine 0.5% and sodium fluorescein 0.25%, which are commonly used in some of the busy clinics when measuring IOP, and the observed swelling was greatest in the central part of the cornea. They further reported that the increase in CCT was around 10 *μ*m but could be as high as 30 *μ*m. The effect of topical anesthetic should be taken into account when measuring CCT in patients with DED, as it can result in overestimation. In the present study, mean CCT value via ultrasonic pachymetry and topical anesthetic eye drop was approximately 6.23 *μ*m higher than that measured via SD-OCT.

Alcaine®, a topical anesthetic eye drop used during ultrasonic pachymetry, includes the preservative benzalkonium chloride (BAC), which is a quaternary ammonium compound shown to hasten tear film drying, exacerbate preexisting DED, and negatively affect the cornea [27]. BAC at a high concentration such as 0.1% was used to induce a dry eye model in animal studies [28]. Many researchers have investigated the acute toxic effects of high concentrations defined as 0.1% of BAC on the ocular surface [29]. A wealth of clinical and experimental evidence supports the notion that the toxic effect BAC has on the ocular surface is primarily concentration dependent. Topical use of BAC can induce ocular surface changes, such as tear film instability, loss of goblet cells, inflammation, epithelial apoptosis, and corneal endothelial cell edema and disappearance, resulting in loss of barrier function and an increase in corneal stroma, which all result in an increase in corneal thickness [30]. BAC can also cause corneal surface epithelial edema and adversely affect the barrier integrity of the corneal epithelium. Currently, BAC is most often used at a concentration of 0.01% in ophthalmic preparations, which appears to be safe in normal healthy individuals. In a recent study, Chen et al. [28] reported that even low concentrations defined as 0.01% of BAC can induce significant corneal stromal alterations. It was reported that in corneal epithelium (the most clinically significant tissue affected in patients with DED) exposed to a desiccating stress in a mouse model of experimentally induced DED the cell proliferation rate and CCT increased significantly [22]. We did not know the safe concentration of BAC in dry eye patients so CCT value differences between these two devices could be related to BAC preservative, which was used during ultrasonic pachymetry.

The tear system is dynamic and blinking plays an important role in distributing tears across the ocular surface. Blinking redistributes tears to the ocular surface from the tear menisci around the upper and lower eyelids and facilitates tear drainage. Due to reflex tearing, the balance between tear secretion and drainage in normal eyes is altered during delayed blinking. During delayed blinking tear menisci increase significantly. Furthermore, the lack of tears in DED patients can result in abnormal tear distribution across the ocular surface and irregular interaction during blinking [31]; however, in DED patients mean precorneal tear film thickness was higher immediately after delayed blinking than after normal blinking [32]. The increase in precorneal tear film during delayed blinking might also indicate that patients with DED may have some ability to produce tears in response to prolonged eye opening. The effect of delayed blinking was also taken into account during the present study while obtaining CCT values, which in all instances occurred immediately after blinking.

In the present study we carefully sought to avoid the effect of systemic drugs on DED because of the fact that systemic drugs (i.e., anticholinergic drugs and antihistaminic drugs) used to treat several diseases can increase the risk of DED. Furthermore, it is known that tear production is reduced in elderly tear-deficient patients that use systemic medications [33] and, as such, the present study aimed to exclude patients using any type of systemic drug. In addition, DED patients with such systemic illnesses as diabetes mellitus were also excluded, as increased cornea thickness has been reported in diabetic patients [34]. Dry eye in diabetics is known to be due to a decrease in corneal sensation or in the relative numbness of the ocular surface. Mean CCT in the corneas of diabetic patients was 27 *μ*m higher than that in nondiabetic controls [34]. It has been posited that increased corneal thickness in diabetics might be due to increased corneal water content, increased corneal dry weight content, or a combination of both. Moreover, mean CCT in diabetics with DED is lower than that in diabetics without DED. To eliminate all confusion regarding the effect of diabetes on CCT in dry eyes, DED patients with diabetes were excluded in the present study. Contact lens wearers were also excluded from the present study because it is known that dry eye is more prevalent in such patients and that long-term contact lens users have lower mean CCT values than those that do not use contact lenses [35]. Pseudoexfoliation (PEX) material can cause a decrease in tear film secretion and disturb tear film stability. Lower CCT values in eyes with PEX material may be a result of a decrease in corneal stromal cell density [36]. As such, we also excluded the patients with PEX material while interpreting the findings.

One of the limitations of the current study is that we were unable to measure the precorneal tear film thickness due to the lack of imaging spectrograph, ultrahigh resolution optical coherence tomography, noninvasive interferometry, and confocal microscopy. However, there is a wide range of mean precorneal tear film thickness values in healthy subjects. There is not a normal level of tear film thickness in agreement present in the literature. Mean central tear film thickness was reported to be 4.79 *μ*m based on ultrahigh resolution OCT [37]. Prydal et al. [38] observed that precorneal tear film thickness was 34–45 *μ*m based on noninvasive interferometry versus 41–46 *μ*m based on confocal microscopy. Wong et al. [39] reported precorneal tear film thickness as 8.0 *μ*m. King-Smith et al. [40] reported the mean precorneal tear film thickness as 2.7 *μ*m using SpectraPro-150 imaging spectrograph.

Another limitation of the present study is that corneal thickness was only measured centrally. According to a recent study, alteration of epithelial thickness caused by DED affects the peripheral corneal epithelium to a greater extent compared to the central region [32]. To determine why the superior epithelium is thinner in dry eyes, the spatial disparity of epithelial thickness in normal eyes should be determined first. The superior corneal epithelium was shown to be significantly thinner than the inferior in normal eyes [41]. This nonuniform thickness profile was suggested to be induced by the friction that results from the mechanical dynamics of blinking [42]. The wider-range movement and vertical traverse of the upper lid rub more of the ocular surface in the superior corneal region. This friction mechanically damages epithelial cells, causing the thinning of the superior epithelium. DED patients usually do not have enough tears for lubrication and it was proposed that the increase in mechanical friction exacerbates epithelial damage and results in even thinner superior epithelium [32].

The prevalence of dry eye is higher in females; our higher number of female participants supports this finding. The high frequency of woman participants in our study made us consider the possible effect of sex hormones on corneal thickness values. The mean CCT value of female patients in our study was approximately 12 *μ*m thinner than the values of our male patients. The cornea of females can be affected by hormonal changes that occur during the monthly menstrual cycle. Indeed, cyclic variations in corneal thickness have been described. Increased corneal thickness measured during ovulation and at the end of the menstrual cycle is based on a previous study by Goldich et al. [43]. Keskin et al. [44] showed a linear correlation between CCT and serum estradiol levels of their patients. They also showed that menopause causes a decrease in central corneal thickness measurements. The mean age of our female patients was 51.7 years, which can be considered as perimenopause period. The reduced corneal thickness values we encountered in our female patients support this finding. Changes in corneal hydration, together with estrogen-mediated changes in corneal cells and corneal extracellular matrix, can all be possible reasons for these corneal thickness changes in females [43, 44]. The effects of hormones on cornea should be taken into consideration while interpreting the central corneal thickness values.

In the present study, mean CCT measured via Cirrus SD-OCT was 6.23 *μ*m less than that measured via ultrasonic pachymetry, which might have been due to manual adjustment of the Cirrus SD-OCT device's scale. The slightest movement of the Cirrus SD-OCT measurement bar has sensitivity of 4 *μ*m. Decreasing the level of sensitivity of the scale to 1 *μ*m may result in exactly the same CCT measurements as obtained via ultrasonic pachymetry. User-dependent CCT differences can be avoided by future software updates to facilitate automated CCT measurements, as used for retinal examination [14]. It is unclear if ultrasonic pachymetry or SD-OCT measurements more accurately indicate the actual corneal thickness in DED patients. Although it is possible that differences in analysis software might account for the discrepancy observed in the present study, the weight of evidence currently available suggests that a systematic difference does exist between SD-OCT and ultrasonic pachymetry and that the discrepancy is unrelated to intraobserver differences in SD-OCT measurements.

In conclusion, the present findings show that in patients with severe DED Cirrus SD-OCT provides reliable intraobserver CCT values and consistent agreement between independently trained observers. Although potential errors can occur with manual measurements, this present finding can inform researchers and clinicians concerning the expected variability when performing this pachymetry technique in severe DED patients. The difference in CCT based on SD-OCT and ultrasonic pachymetry measurement should always be a consideration when interpreting CCT values in severe DED patients.
